# Genome Sequence of *Mycoplasma hyorhinis* Isolated from Cell Cultures

**DOI:** 10.1128/genomeA.01119-16

**Published:** 2016-10-13

**Authors:** Samuel Paulo Cibulski, Franciele Maboni Siqueira, Thais Fumaco Teixeira, Fabiana Quoos Mayer, Luiz Gonzaga Almeida, Paulo Michel Roehe

**Affiliations:** a Faculdade de Veterinária, Laboratório de Virologia, Universidade Federal do Rio Grande do Sul, Porto Alegre, Rio Grande do Sul, Brazil; b Departamento de Microbiologia Imunologia e Parasitologia, Laboratório de Virologia, Universidade Federal do Rio Grande do Sul, Porto Alegre, Rio Grande do Sul, Brazil; c Centro de Biotecnologia, Universidade Federal do Rio Grande do Sul, Porto Alegre, Rio Grande do Sul, Brazil; d Laboratório de Biologia Molecular, Instituto de Pesquisas Veterinárias Desidério Finamor (IPVDF), Fundação Estadual de Pesquisa Agropecuária Eldorado do Sul, Rio Grande do Sul, Brazil; e Laboratório de Bioinformática, Laboratório Nacional de Computação Científica (LNCC) Petrópolis, Rio de Janeiro, Brazil

## Abstract

Mycoplasmas are major contaminants of mammalian cell cultures. Here, the complete genome sequence of *Mycoplasma hyorhinis* recovered from Madin-Darby bovine kidney (MDBK) cells is reported.

## GENOME ANNOUNCEMENT

Mycoplasma contamination is a major challenge to cell and tissue culture; in one study, more than 35% of the tested cell lines were found to be contaminated (1). Mycoplasma competes with host cells for biosynthetic precursors and nutrients, altering cellular metabolism. Furthermore, contamination can alter gene expression (2) and exert significant effects on cultured immune cells (3). Eventually, contamination may compromise the results of biological assays or affect its interpretation. Moreover, as mycoplasmas may also cause cytopathic effects (CPE) resembling those of viruses, many investigators have mistaken cytolytic mycoplasmas for viruses.

In swine, *Mycoplasma hyorhinis* is associated with respiratory tract infections and arthritis (4). *M. hyorhinis* is the *Mycoplasma* species most often detected as a contaminant in cell cultures (5). In addition, chronic *M. hyorhinis* infection has been associated with malignant transformation (5, 6). Here, the complete genome sequence of *M. hyorhinis*, recovered from a Madin-Darby bovine kidney (MDBK, originally ATCC CCL-22) cell lineage, is reported.

As part of our bovine herpesviruses genome sequencing studies in the MDBK cell line, whole-genome sequencing disclosed an inadvertent mycoplasma coculture. Virus was cultured in MDBK, clarified by low-speed centrifugation, filtered (0.45-µm-diameter pore), and ultracentrifuged (7). DNA was extracted according to a universal phenol-chloroform protocol. DNA libraries were prepared with a Nextera kit. Next-generation sequencing was performed on Illumina MiSeq equipment (Illumina), with a 500-cycle kit (version 2) to generate 2 × 250 paired-end reads.

Reads were imported into the Geneious software (version 8.1) and trimmed. Assembly of the *M. hyorhinis* genome was then accomplished by template-assisted assembly, in which trimmed reads were mapped to the reference *M. hyorhinis* genome (*M. hyorhinis* strain DBS 1050, accession no. CP006849) with the Geneious software and *de novo* assembled with SPAdes 3.6 (8). The genome was then assembled to a single circular contig, annotated, and each gene was manually curated using the SABIA tool (9).

The complete genome of *M. hyorhinis*, named MDBK/IPV, consists of a circular chromosome of 837,377 bp, with an overall G+C content of 25.9%. Among 752 potential protein-coding genes, 83% encode proteins with assigned functional roles; the remaining 17% encode hypothetical proteins.

The genome of *M. hyorhinis* MDBK/IPV includes 32 tRNA genes; only a single copy of the 16S-23S rRNA operon can be found. The 5S rRNA operon is separated from the 16S-23S rRNA operon. Additionally, 43 transposase gene copies were mapped in the genome, most of them truncated or inactivated, with multiple frameshift mutations.

*M. hyorhinis* contains a genetic system that allows for surface antigenic variations at high frequency. The variable-surface lipoprotein (*vlp*) locus contains up to seven distinct single-copy *vlp* genes (10). The size variation of Vlp products results from the insertion or deletion of tandemly repeated intragenic sequences that expand or contract the surface Vlp C-terminal region (11). The *vlp* locus constitution is varied among *M. hyorhinis* strains, with insertion sequences (IS) that are often detected in all *vlp* loci (12–15). The *M. hyorhinis* MDBK/IPV *vlp* locus is represented by six *vlp* genes divided by two degenerate IS elements: 5′*-vlpC*-*vlpB-*IS-*vlpA*-IS-*vlpF*-*vlpE*-*vlpG*-3′***.***

### Accession number(s).

The complete genome sequence of *Mycoplasma hyorhinis* strain MDBK/IPV has been deposited at GenBank under the accession no. CP016817.
